# Antimicrobial resistance mechanisms and potential synthetic treatments

**DOI:** 10.4155/fsoa-2017-0109

**Published:** 2018-02-05

**Authors:** Junaid Ali, Qasim A. Rafiq, Elizabeth Ratcliffe

**Affiliations:** 1 Department of Chemical Engineering, Centre for Biological Engineering, Loughborough University, Loughborough, Leicestershire, LE11 3TU, UK; 2 Advanced Centre for Biochemical Engineering, Department of Biochemical Engineering, University College London, London, WC1E 6BT, UK

**Keywords:** antibiotics, antimicrobial resistance, infectious agents, molecular biology, re-sensitizing, resistance mechanisms

## Abstract

Since the discovery of antibiotics by Sir Alexander Fleming they have been used throughout medicine and play a vital role in combating microorganisms. However, with their vast use, development of resistance has become more prevalent and their use is currently under threat. Antibiotic resistance poses a global threat to human and animal health, with many bacterial species having developed some form of resistance and in some cases within a year of first exposure to antimicrobial agents. This review aims to examine some of the mechanisms behind resistance. Additionally, re-engineering organisms, re-sensitizing bacteria to antibiotics and gene-editing techniques such as the clustered regularly interspaced short palindromic repeats-Cas9 system are providing novel approaches to combat bacterial resistance. To that extent, we have reviewed some of these novel and innovative technologies.

## Lay abstract

In 1928, penicillin was discovered, changing the field of modern medicine as it provided an opportunity to treat microbial infections. Since then, microorganisms such as bacteria have evolved and now have the ability to resist a wide variety of agents that might otherwise prevent their growth. By 2050, it is estimated that around 10 million lives each year will be lost due to these bacteria. This article provides an insight into how bacteria resist antibiotics and potential new methods of treating these organisms.

Antibiotics are commonly used in the treatment of bacterial, yeast and parasitic infections [1]. They are used to inhibit cell wall, protein and nucleic acid synthesis while inhibiting the growth and proliferation of microbes, and have been used for decades to combat infections in humans and animals. The first ‘modern day’ antibiotic, penicillin, discovered by Alexander Fleming in 1928, remains widely used to the present day. After its discovery, it was complemented by a series of other antibiotics such as the sulfonamides and aminoglycosides, examples of which include sulfamethazole and gentamicin, respectively. Antibiotics have revolutionized medicine, treating infections that were once fatal. However, with their vast use throughout the world, resistance to antibiotics has developed with some resistance emerging within a year after the introduction of the antibiotic and many others developing resistance within half a decade [2]. One of the most important documents within the field of antibiotic resistance is the O'Neill report which encompasses all aspects of reducing its potential future effect and includes raising awareness, improving hygiene to prevent infection spreading, reduction in unnecessary use of antibiotics, improved surveillance of drug resistance and promotion of new diagnostics. All these recommendations are being made to make sure antibiotic treatment is still possible [3]. There is growing concern that resistance to modern antibiotics will mean that bacterial infections become untreatable unless novel technologies are developed.

Around 50,000 lives per year are thought to be lost due to antimicrobial resistance (AMR) infections within the USA and Europe [4]. AMR infections are projected to cause 10 million deaths per annum by 2050, with 4.1 million and 4.7 million deaths due to AMR infections in Africa and Asia respectively, at a cost of $100 trillion [3]. The WHO estimates that antibiotic microbial resistance (AMR) costs >$1.5 billion every year in healthcare expenses in the EU alone (WHO 2016). The aim of this paper is to review some of the mechanisms microorganisms use to resist antibiotics, and examine molecular mechanisms to target AMR.

## Antimicrobial resistance mechanisms

It may be argued that antibiotics are one of the most successful therapies in modern medicine for treating bacterial infections. With the recent rise in AMR, understanding the mechanisms by which bacteria resist antibiotics will become critical to solving the crisis [5]. Misuse of antibiotics may contribute to the development of resistant bacteria, an incomplete course of antibiotics risks not entirely eradicating the colony thus allowing the development of resistant bacteria [6,7]. Additionally, the US FDA explains that skipping doses, saving, and sharing antibiotics allow resistant bacteria to form (FDA 2016).

**Figure 1.**
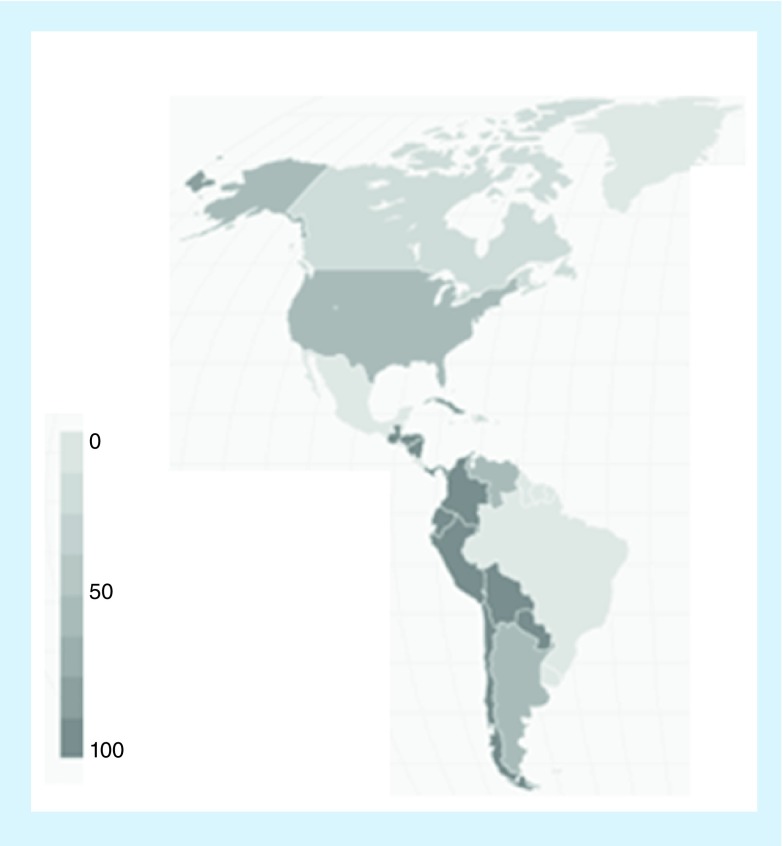
**The percentage of *Staphylococcus aureus* that is methicillin resistant in North and South America.** Data taken from [12].

Recently, there has been the emergence of ‘super resistant bugs’, multidrug-resistant forms with an increased level of resistance or those that are resistant to multiple antibiotics such as methicillin-resistant *Staphylococcus aureus* (MRSA) which is estimated to cause over 11,000 deaths per year [8]. Around 60% of all *S. aureus* infections are methicillin resistant, and around 7 per 1000 hospital admissions are treated for MRSA. Figure 1 shows the percentage of *S. aureus* that is methicillin resistant in North and South America. A further example is the multidrug-resistant *Salmonella enterica* which is resistant to five antibiotics; ampicillin, chloramphenicol, streptomycin, tetracycline and methoxazole. It is estimated that there are over 150,000 deaths per year due to these infections [9–11]. Unless novel methods are used to tackle this threat, the cost to healthcare services will increase as will the mortality rate, thus highlighting the need for novel AMR therapies. With 80% of antibiotics sold in the USA of which 70% of these are prescribed incorrectly, and up to half of all antibiotics used in humans used incorrectly, it seems obvious why drug resistance is becoming such a prevalent threat. Resistance can occur through a reduction in the drug permeability, biofilm formation reducing the susceptibility to antibiotic activity, active efflux pumps and others outlined in this section.

### Intrinsic resistance

Environmental changes, such as radiation, changes in light or pH can all contribute to resistant bacteria and have been reviewed thoroughly [13–16]. However, a consideration must also be given to the internal intrinsic resistance that bacteria naturally possess. Intrinsic resistance usually utilizes enzymes to destroy the drug or prevent intracellular drug binding within the target organism. The innate ability of bacteria to resist antibiotics is known as ‘insensitivity’ as organisms have not been exposed to that drug but still have a level of resistance [17]. Different species and strains all possess their own unique individual genotype and phenotype to AMR as outlined in Table 1.

**Table 1.** **A table to show the natural resistance and its mechanism for specific bacteria.**

**Intrinsic resistance against antimicrobial agents**	**Organism**	**Mechanism**	**Ref.**
Aminoglycoside	Anaerobic bacteria	No oxidative metabolism for uptake of antibiotic	[18]

Chloramphenicol	Lactobacilli and leuconostoc	Lack of appropriate cell wall precursor target to allow binding and inhibit cell-wall synthesis	[19]

Metronidazole	Aerobic bacteria	Unable to reduce drug to its active form	[20]

Vancomycin	Gram-negative bacteria	Outer membrane is impermeable to large glycopeptide	[21]

Vancomycin	Enterococci	Lack of sufficient oxidative metabolism to drive uptake of glycopeptide antibiotics	[22]

β-lactams	Enterococci	Lack of penicillin binding proteins that effectively bind and are inhibited	

β-lactams	Gram-positive bacteria	Lack of penicillin-binding proteins that bind and are inhibited by the antibiotic	[23]

β-lactamases	Stenotrophomonas, maltophilia	Antimicrobial agents that production of enzymes (β-lactamases) that destroy imipenem before the drug can reach the PBP targets	

Ampicillin	*Klebsiella*	Produces β-lactamase that destroy drug before it reaches penicillin-binding protein targets	[24]

Carbenicillin	*Pseudomonas aeruginosa*	Lack of uptake causing a lack of intracellular concentration and an inability of antibiotics to achieve effective concentration	[25]

Bacterial lipocalins are a small group of proteins that are widely conserved in both gram-positive and -negative organisms, and confer resistance against hydrophobic antibiotics. However, their physiological role remains unclear. With their discovery coming <25 years ago, much remain unknown about their potential as an AMR mechanism. However, one study used *Pseudomonas aeruginosa* PAO1 and introduced stress via antibiotic exposure. Transcription of *BcnA*, a gene conferring antibiotic resistance to hydrophobic antibiotics, was increased, showing that it may play a role in resistance. *In vitro* survival of *P. aeruginosa* PAO1 was significantly increased when treated with *BcnA* with a significant decrease in sensitivity when compared with polymyxin B treatment p < 0.001. As gene transcription was significantly increased under antibiotic stress, this showed a level of intrinsic resistance [26].

### Mutation

Since the mid-20th century, multidrug-resistant pathogens have had a clinical relevance, and can be attributed to mutations in the bacteria themselves [27]. Recently, with the improvement of molecular techniques, mutations have become more apparent and easier to detect.

Random point mutations can occur in any species and have recently been documented in *Helicobacter pylori* [28–30]. Mutations within the 23 s rRNA are of particular interest as within it lies the most common binding site for antibiotics to inhibit transcription and translation. Clarithromycin resistance or mutations in the 23 s rRNA gene of *H. Pylori* may prevent the treatment of pneumonia and skin infections [31–34]. Interestingly, mutations in the 23 s rRNA in *Staphylococcal* species are associated with reduced susceptibility to linezolid. Extensive clinical use of linezolid has led to resistance selection in *S. aureus* and *Streptococus pneumoniae;* however, it is still effective in >98% of *Staphylococcus* species [35].

**Figure 2.**
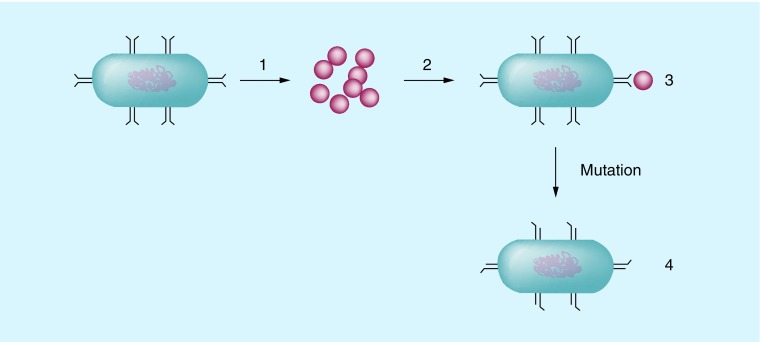
**A diagram to show how mutations lead to antibiotics being unable to bind to their target.** **(1)** A wild-type bacteria. **(2)** Antibiotics that would bind to and destroy the target bacteria. **(3)** The antibiotic is able to bind to and destroy the target wild-type bacteria. **(4)** After mutation occurs, the binding site is altered and the antibiotic is unable to bind to the mutant bacteria and is unable to kill it. These bacteria will proliferate creating a new resistant colony.

Roux *et al*. examined the effect *in vivo* of mice with gastrointestinal *P. aeruginosa* infections with an inactivation of *ampC, aph, mexA* and *oprM*. The fitness of mice was significantly reduced in the mutants compared with the wild-type in both the GI model and lung infection model. Interestingly in their paper, they explain that clinical *pseudomonas* isolates commonly have an *OprD* mutation which is the main mechanism of carbapenem resistance. They state that a consequence of AMR could be enhanced fitness of pathogens and that reducing antibiotic use may not reduce the number of resistant strains but reduce the selective pressure from antibiotic-resistant strains. Figure 2 below shows the mechanism as to how mutations in bacteria lead to their resistance against antimicrobials.

### Inactivation of antibiotics

Bacterial enzymes have been shown to add chemical groups to vulnerable sites on the antibiotic molecule preventing the antibiotic from binding to its original target [36]. Within the structure of an antibiotic, hydroxyl and amides groups can easily be changed by hydrolysis [37]. Moreover, acetyl, phosphate and nucleotide groups can be added to the antibiotics inactivating them [38].

*Staphylococcus aureus* has shown resistance to penicillin which is mediated by the *blaZ* gene that codes for β-lactamase [39]. β-lactam antibiotics inhibit the biosynthesis of the bacterial cell wall by preventing the cross links which form the wall, and weaken it leaving bacteria unable to proliferate [40]. The β-lactamase enzyme is synthesized when *Staphylococci* are exposed to β-lactam antibiotics and hydrolyses the ring leaving the antibiotic inactivated and unable to destroy the bacteria [41].

### Horizontal gene transfer

For microbial species to maintain resistance to antibiotics, they must not only pass resistance genes to their progeny but also have the ability to transfer genes between species, known as horizontal gene transfer [42]. There are three types of horizontal gene transfer which include: the AMR gene being associated with mobile genetic elements such as mobile introns; loss of synteny (genetic loci) of the insertion site in the host; and acquiring an AMR gene through gene transfer [43,44]. Multiple copies of resistant genes give bacteria the best possible chance to avoid antibiotics. An example can be found in Gram-positive bacteria to provide linezolid resistance, where there are six copies of the gene *23 s rRNA* which play an important role in AMR [45]. However, this has the metabolic burden of producing extra proteins [46]. Vonwintersdorff *et al*. recently reviewed horizontal gene transfer more thoroughly [47].

In addition to the mobile genetic elements, the integrons, a type of transposon, can be transferred to other bacteria allowing bacteria to evolve due to the acquisition of new genes. Theoretically, DNA can be randomly inserted onto the nonhomologous end of a DNA sequence; however, the likelihood and frequency of this is extremely low. Natural transformation may also occur with AMR genes integrated into the bacterial genome [48]. In their paper they also explored the transfer of proteobacterial genes, Cmx and LmrA.

One key recent study examined the role that plasmids play in horizontal gene transfer with stool samples collected from hospitalized patients and compared with healthy volunteers. From the patients, 46 antibiotic resistance genes were identified and categorized into 25 resistance gene types. There were a number of genes that were found common between the patients but most interestingly, there was a resistance gene found in the healthy volunteer who had no exposure to antibiotics for over 3 years and the authors were unable to trace the origin of the gene or the transfer due to the lack of samples. The genes found in the patients have been reported in foods and animals showing the extraordinary transfer of genes throughout the globe [49].

### Efflux pumps, biofilm resistance & quorum sensing

Biofilm formation occurs naturally through quorum sensing by the detection of extracellular autoinducers. The increased cell density in a biofilm enhances its ability to resist antibiotics compared with planktonic cells. The improvement in resistance is due to the reduced ability to diffuse through the matrix of the biofilm, quorum sending and efflux pump expression. The efflux pumps, known to be one of the major causes of resistance, improve antibiotic tolerance in biofilms which is due to their ability to block membrane channels, alter the chemical design of antibiotics and prevent multicomponent pumps. It is known that *P. aeruginosa* possesses intrinsic resistance to a number of antibiotics such as β lactams and many others. One study, named an efflux pump in pseudomonas biofilms, PA1874–1877, which was shown to be expressed to a greater degree when in biofilm culture compared with planktonic culture. The pump was shown to be involved with biofilm exposure to tobramycin, gentamicin and ciprofloxacin [50]. It must be noted that to date, there seems to be a lack of research into the role of efflux pumps when concerned with biofilm resistance, although studies conducted show that targeting the efflux pumps could be the key to unlocking their role in biofilm resistance.

Cell–cell communication (quorum sending) is vital for all cells and is one method of biofilm formation. By inhibiting this communication between cells, there may be a way to prevent the spread of AMR. One quorum-sensing inhibitor ‘HAM’, which affects quorum sensing through the thrombin receptor activated peptides (TraP) receptor and prevents communication, was tested to determine its effect against *S. aureus* biofilms. The study showed that HAM increased susceptibility of *S. aureus* biofilms against a variety of antibiotic classes indicating its potential. Additionally, the susceptibility of HAM was tested against mutations on genes involved in quorum sensing. It was shown that only those with no mutations were affected indicating that HAM only affects biofilm susceptibility through the *S. aureus* quorum-sensing system [51].

## Molecular applications against AMR bacteria

As with many current antibiotic alternatives, treatments may not necessarily be used to treat patients, but used for *in vitro* or *in vivo* research. Some of the treatments reviewed in this manuscript are used to prevent the infection of nosocomial infection rather than for their direct treatment *in vivo*. Synthetic biologists use engineering methods to design, construct and produce new products and pathways with enhanced qualities. Using synthetic biology for designer cells has vastly improved capabilities to diagnose, prevent and treat diseases. Because of the ability of synthetic biology to engineer advantageous products, it may be in a good position to tackle the growing issue of AMR [52]. Recently, the potential of synthetic biology for the therapeutic and diagnostic aspects against antimicrobial-resistant bacteria was reviewed; however, our paper aims to review some of the potential treatments such using clustered regularly interspaced short palindromic repeats (CRISPR) and re-sensitizing bacteria to antibiotics [53].

Production of novel antimicrobial agents is decreasing, possibly due to FDA restrictions such as the need for purity of products. The requirements and standards set are so great and with drugs commonly withdrawn at any time during their development, the risk to reward ratio can seem too great for some [54]. With advancement in both knowledge and engineering capabilities, studies have now moved toward tuning gene regulation and development of new methods to tackle AMR [55]. Genes that code for AMR are known; however, much of the current research focuses on targeting genes that are essential to bacterial life of which there are around 300 [56].

### CRISPR-Cas9 system

There have been many advances in using the CRISPR system for use in AMR research, which builds on previous work using zinc finger nucleases [57]. CRISPR uses a gRNA and a Cas9 protein-modified sequence that needs to be repaired [58]. It is a natural adaptive immune mechanism and is able to cleave foreign DNA. After its initial identification, it became clear it had a use in human therapy and more specifically targeting AMR. There are three types of CRISPR-Cas9 systems, type I cleave and degrade DNA, type II cleave DNA and type III, cleave DNA and RNA. In 2002, it was revealed that CRISPR loci can be transcribed into small RNAs and that the *cas* genes were identified as a part of the same family of the CRISPR loci [59]. Introducing lesions to reprogram the bacterial genome through the eradication of resistance genes using the CRISPR-Cas9 system offers a method to aid in treatment of AMR bacteria [60].

In 2014, two key papers were published that looked at CRISPR-Cas delivery of antimicrobials. Bikard *et al*. (2014) looked at phage encapsidation against *S. aureus* using phagemids that encoded spacers to target antibiotic resistance genes and delivery of the genes significantly sensitized the bacteria *in vitro*. They extended their study to an *in vivo* model where mouse skin was infected with *S. aureus* and phagemids using the CRISPR delivery system and it was compared with topical treatments. Their study found the phagemids significantly reduced bacterial infection and while it was found to be similar to standard topical treatments, it did not fully eliminate the bacteria, unlike streptomycin treatment (10 μg/ml) [61]. The second paper, published by different authors, used a conjugative plasmid carried by *Escherichia coli*, and when contact with the desired strain was made, the plasmid conjugated with the host. However, this method was found to be inefficient as host organisms were incorrectly chosen. Their experiment was redesigned for m13-based phagemids that encode specific genes. It was found to deliver the packaged DNA more efficiently although it was significantly less potent than antibiotics [62].

Kim *et al*. then applied the CRISPR-Cas9 system to extended-spectrum β-lactamase (ESBL)-producing *E. coli* [63]. They targeted sequences that were conserved in ESBL mutants to restore sensitivity to antibiotics in these bacteria. It was shown that these target sequences can be exploited to re-sensitize multidrug-resistant cells in which resistance is mediated by those genes that are not the target of the CRISPR/Cas9 system, but by genes that are present on the same plasmid as target genes. More than 99% of the cells were killed that were treated with the ESBL plasmid, further highlighting the potential that CRISPR has against AMR [63].

In addition to the use of CRISPR for the delivery of antimicrobials and re-sensitization of bacteria, other studies have been performed to detect the presence of resistance genes using the technique. Using the Cas9 protein, plasmids were cut into their linear form allowing gene detection. However, not all plasmids were cut which allowed the authors to correspond to other sizes of known plasmids and allowed them to detect bacterial resistance genes although sequencing them may have been a more appropriate option [64].

As well as targeting resistant bacteria on hospital surfaces, using the CRISPR-Cas9 system to de-colonize patients with resistant bacteria, could be used as the system is well understood and is highly specific, therefore reducing the chance of widespread resistance. However, a limitation of the system is the delivery of the CRISPR-Cas9 components. Within a cell culture model, there are established methods to deliver plasmids through the cell membrane. Delivery *in vivo* represents greater challenges, the inability to penetrate membranes, low serum tolerance, large size and negative charge. Additionally, large DNA packaging and large-scale vector production perhaps represent a greater challenge [65]. In addition, when using nonviral vectors, there has been a low delivery efficiency which again represents a significant barrier to its use. This novel technique may be more useful in other areas rather than the direct treatment of patients as so far methods generally are less potent that antibiotics. CRISPR has been used in a variety of areas in research and it has been specifically reviewed more comprehensively for its uses in genome editing [66]. However, with the use of CRISPR for gene editing, it will be possible to remove resistance genes from AMR bacteria thereby reducing the number of resistant organisms and reducing their potential for human infection.

### Re-sensitizing bacteria to antibiotics

One novel possibility in the treatment of resistant bacteria is the potential to re-sensitize resistant bacteria to antibiotics. While it may be a future possibility that this is used *in vivo*, the more realistic immediate potential for this treatment would be against nosocomial pathogens or pathogens on hospital surfaces and medical devices.

Re-sensitizing bacteria to antibiotics after resistance has developed, may be a valid method of treating AMR; however, there are a limited number of studies that have adopted this approach [67]. Goh *et al*. recently targeted AMR genes with antisense agents to reduce their expression and consequently restore sensitivity. They found that antisense peptide nucleic acids, which are gene specific and inhibit a group of bacteria with specific sequences, were effectively able to inhibit the growth of MRSA by 66% at 2.5 μM of anti-*mec* peptide nucleic acid (PNA) and 92% of methicillin-resistant *Staphylococcus pseudintermedius* [68].

Although re-sensitizing bacteria to antibiotics may prove to be an invaluable approach, further studies around the underpinning biology are required. An alternative method to address AMR is to boost metabolism. One study tried to stimulate central metabolic pathways to enable aminoglycosides to eradicate *E. coli* and *S. aureus* biofilms [69]. The addition of exogenous metabolites such as glucose, fructose and mannitol boosted the bactericidal effects against the biofilms and offers the potential to address AMR by restoring metabolic deficiencies. Individually gentamicin was supplemented with glucose, mannitol, pyruvate and fructose, and each combination reduced persisters by three orders of magnitude 2 h after treatment [70].

There have been a small group of molecules known as 2-Aminoimidazole which have the ability to re-sensitize bacteria to antibiotics and have the potential to be used in treating persistent dermal infections. *In vitro* experiments using *S. aureus* and a mixture of penicillin/streptomycin showed a reduction from 7.84 ± 0.56 log colony-forming unit/cm^2^ to 6.46 ± 0.07. However, when 2AI-H10 was used in combination, there was a 4log decrease in colony-forming unit/ml (p < 0.007) thus effectively re-sensitizing *S. aureus* to antibiotics. *In vivo* applications using a porcine skin model and the scratch closure assay were shown to cause no abnormalities or immune reactions when a scratch closure assay was performed [71].

Spero therapeutics recently developed an antibiotic adjuvant to tackle nosocomial infections. Their product, SPR741, was used in combination with antibiotics and was shown to increase in potency against a spectrum of Gram-negative organisms. Over two dozen antibiotics have had their spectrum expanded when used in combination with the product. With future products in the pipeline, this technology may enhance the lifespan of antimicrobial agents [72].

## Conclusion

It is clear that AMR currently represents a significant problem with statistics showing that within the next three decades there will have been a substantial number of mortalities and huge capital lost through the production of antibiotics and man hours spent developing treatments.

The challenge will be the implementation of novel technologies to treat clinically relevant infections. CRISPR has been used in many areas of science and may also have a part to play in the fight against bacteria showing AMR with recent advances made by eradicating resistance genes. Additionally, work by Draughn *et al*. showed that re-sensitizing bacteria to antibiotics shows a significant promise to combating AMR bacteria as they were effectively able to reduce *S. aureus* by 4log. Synthetic biology may provide a valuable approach to target resistant bacteria by re-sensitizing bacteria to antibiotics and through the use of synthetic antimicrobial peptides, as *in vitro* studies to date have shown promising results [71].

Understanding fully the mechanisms of resistance may be key to unlocking its treatment; however, any advances made may always be under threat as horizontal gene transfer has shown the ability to pass resistance genes between species of bacteria but also between humans/animals and foods highlighting its strong influence.

## Future perspective

It may be argued that one of the greatest challenges that faces our population today is the emergence of AMR. D'Costa reported the ancient nature of AMR and with the development of new resistant microorganisms, and the recent rise in their numbers, novel techniques to tackle them are needed [17]. Grants such as the longitude prize and the significant amount of money being invested into the focus of AMR show that there is hope that one day, novel therapies may be able to tackle the problem. However, their use must be controlled so that within another century similar problems are not repeated. Promising techniques such as CRISPR which allow the novel delivery of therapeutics as well as targeting mutant sequences in microorganisms have been developed. Additionally, re-sensitizing bacteria to antibiotics which they were once resistant to, offers much promise to tackle nosocomial infections that cause many mortalities. In addition, the research into the mechanisms of resistance offers the hope of tackling the problem such as understanding their intrinsic resistance as well as environmental causes.

**Executive summary**

**Background**

- It is clear that antimicrobial resistance currently represents a significant problem.
- Understanding fully the mechanisms of resistance may be key to treatment.
- We review some novel and innovative technologies in this area.

**Antimicrobial resistance mechanisms**

- One of the key methods to unlocking antimicrobial resistance will be to understand the mechanisms as to how microorganisms develop resistance.
- Cell–cell communication and the transfer of resistant genes between organisms represent a greater level of resistance by the bacteria.
- Efflux pumps and biofilm formation from the bacteria provide an added level of their resistant properties.

**Molecular applications against antimicrobial resistance bacteria**

- One of the biggest leaps forward in science came with the clustered regularly interspaced short palindromic repeats-Cas9 system which has been exploited for gene editing.
- Clustered regularly interspaced short palindromic repeats-cas9 is a simple and versatile method which uses two enzymes, to cut DNA and replace with a new sequence.
- This technique has been exploited to tackle antimicrobial resistance as well as using it for delivery of antimicrobials.
